# Systematic Review of Alexithymia in the Population of Hemodialysis Patients

**DOI:** 10.3390/jcm10132862

**Published:** 2021-06-28

**Authors:** Đorđe Pojatić, Ivana Tolj, Davorin Pezerović, Dunja Degmečić

**Affiliations:** 1 Faculty of Medicine Osijek, Josip Juraj Strossmayer University of Osijek, 31000 Osijek, Croatia; djolepojatic@gmail.com (Đ.P.); ivanatolj5@gmail.com (I.T.); 2 Faculty of Dental Medicine and Health Osijek, Josip Juraj Strossmayer University of Osijek, 31000 Osijek, Croatia; davorin.pezerovic@icloud.com; 3 Department of Internal Medicine, General County Hospital Vinkovci, 32100 Vinkovci, Croatia; 4 Department of Internal Medicine, University Hospital Osijek, 31000 Osijek, Croatia; 5 Department of Psychiatry, University Hospital Osijek, 31000 Osijek, Croatia

**Keywords:** alexithymia, hemodialysis patient, chronic kidney disease

## Abstract

Alexithymia is a construct defined as the inability to differentiate between emotional experiences and bodily sensations. According to existing knowledge, alexithymia may have a major effect on the process of treatment and the outcome of the hemodialysis disease. The objective of this literature review was to determine the significance that alexithymia has for compliance and variables of clinical and mental health in the population of hemodialysis patients. For the above purpose, bibliographic databases “MEDLINE” and “Web of Science” were searched. The matrix method was used in analysis of articles. Searching both databases resulted in 248 articles. After applying exclusion and inclusion criteria, we included results of 13 articles in the literature review. The results of the search are findings regarding the prevalence and correlation of alexithymia with variables of clinical and mental health in hemodialysis patients. Alexithymia is significantly more common in the population of hemodialysis patients, and it has a negative effect on their mental and somatic health. Alexithymia levels in hemodialysis patients are more pronounced in cases where there is a greater number of comorbidities. Alexithymia is the predictor of high mortality rate in the population of hemodialysis patients, independent of other comorbidities.

## 1. Introduction

End-stage chronic kidney disease (CKD) is a disease defined as a state of reduced glomerular filtration rate with a creatinine clearance lower than 15 mL/min/1.73 m^2^. In patients with CKD, there are signs of electrolyte imbalance and fluid overload with oliguria or anuria. With the aim of surviving and ensuring good-quality continuation of life, patients with this stage of CKD commence treatment with hemodialysis, a treatment method based on the physical process of diffusion of metabolic waste through the semipermeable membrane of the dialyzer.

There is no clear consensus about dialysis adequacy, but according to the most recent guidelines, to be effective, hemodialysis should be performed several times a week for several hours [1]. A patient on hemodialysis (an HD patient) is recommended to follow a strict diet plan and reduce fluid intake; the time required for the treatment also represents a physical and psychological stressor. In addition to the above, during their stay in hemodialysis units, HD patients often witness emergencies regarding other HD patients, resuscitation with fatal outcome and they themselves occasionally suffer from hemodialysis complications [2]. All of the above causes great stress which HD patients have to cope with on their own, using their own intrapsychic capacities. Due to the high level of stressors and increased levels of caused frustrations in HD patients, with time, such patients start to respond with immature defense mechanisms of ego, such as denial (which is the most expressed one) [3]. According to certain studies, negation of stress leads to the development of alexithymia [4]. Alexithymia is a construct, a notion that describes the inability to differentiate between bodily sensations and emotions, difficulty in describing emotions to others as well as a cognitive style guided by the objective, factual state [5]. High levels of alexithymia are a predisposing factor for various psychiatric and somatic disorders [6]. The mechanisms by means of which the reduced ability to differentiate between emotions and bodily sensations leads to the development of somatic disorders have not been fully explained, but the potential overactivation of the autonomic nervous system is considered in situations where the alexithymiac interprets a negative emotion as a negative bodily sensation, which over time leads to the development of psychosomatic disorders [7].

Alexithymia may be primary, i.e., conditional on characteristics of the individual’s personality, or secondary, i.e., a consequence of continuous psychosocial stress and somatic disorders [8]. Secondary alexithymia occurs in individuals facing consequences of physical disorders, and is a factor that significantly reduces the ability to take regular care of oneself, to comply with the diet plan and to react to physical manifestations of disease in a timely fashion [9,10]. Thus, in the general population, alexithymia is associated with more frequent consumption of unhealthy foods and a lack of self-control in food and fluid intake, which can be a very dangerous combination for HD patients, as uncontrolled and excessive consumption of fresh vegetables or fruits could lead to life-threatening hyperkalemia [11] Because HD patients have difficulties distinguishing between emotions and bodily sensations, it is reasonable to assume that they may be late in understanding they are in situations that require emergency dialysis, such as shortness of breath due to hypervolemia [8] In the long run, junk food consumption, which is much more frequent in alexithymic individuals in the general population, could lead to more rapid development of cardiovascular problems, which HD patients are already more prone to due to high levels of oxidative stress caused by CKD [12].

According to previous research, HD patients are more prone to somatization and projection than the general population. Somatization is an immature defense mechanism in which frustration-induced discomfort manifests through feeling of somatic symptoms, and projection occurs when HD patients attribute their own unacceptable thoughts and desires to others [13] Given that immature defenses have been associated with high levels of alexithymia in studies conducted both on specific clinical samples and in the general population, it has become clear that alexithymia is a construct that can make communication with medical staff difficult in a number of different ways [14].

For the above reasons, alexithymiacs may continuously report symptoms of “non-existent diseases or fail to report a significant physical problem to the competent medical staff, which makes them fit for development of complications of bodily disorders” [7].

Due to the great importance of alexithymia and its potential consequences on physical health, and because of the wide limitations and obligations of HD patients, alexithymia may have an exceptionally negative effect on mental health, compliance with treatment and the clinical status of HD patients. Since there are no literature reviews concerning this significant matter, we believe that the questions of significance of alexithymia in HD patients and its relationship with other factors of psychosocial and physical health must be answered.

Therefore, our aim with this review article was to answer questions concerning correlation and influence of alexithymia on a somatic and mental health of patients, on a chronic hemodialysis treatment program by searching through cross-sectional and longitudinal studies.

## 2. Materials and Methods

The matrix method was used for searching for articles in the databases. The matrix method represents a clear, organized plan for searching the literature using keywords with the aim of finding relevant articles, which are then organized in a table for the purpose of adequate overview and insight into data. Data on an article, including objectives, methods, results, strengths and weaknesses of individual studies, represent a guide for organizing information from the literature into chapters of the literature review [15]. In this literature review, cross-sectional and longitudinal studies were included, which aimed to investigate prevalence and correlations of alexithymia with factors of mental and somatic health in patients in chronic hemodialysis programs compared to HD patients which were not alexithymic. The systematic literature review included original articles which were conducted in the period from 1989 to 2021, but no restrictions about the publication date were imposed. We were unable to include articles that were not published in English. Studies conducted on children and adolescents under the age of 18 were not the subject of our search.

The databases that were searched were the bibliographic databases MEDLINE, National Institutes of Health, Bethesda, Maryland, USA and Web of Science, Clarivate Analytics, Philadelphia, United States and the last search was run on 20 April 2021. We used the keywords that were selected via the SPICE method [16]. The keywords “hemodialysis”, “hemodialysis patients”, “end stage renal disease” and “end stage kidney disease” were combined with the term “alexithymia”.

Selection of studies for further data analysis was done by mutual consensus of the authors referring to earlier defined inclusive research criteria. Studies that were obtained by searching the above databases, but whose titles and abstracts did not contain the above keywords, were excluded from further study. Studies comparing HD patients with patients undergoing peritoneal dialysis or with former HD patients who received transplants were included in the matrix if the findings regarding alexithymia in HD patients in such studies were obtained on an adequate sample of HD patients, i.e., if they had sufficient statistical power or provided useful qualitative results. The matrix method was used to extract data for each study individually. Data of participants, study design, outcomes, limitations and strengths of studies were all arranged (see Table 1), enabling a better insight on the quality of the data of selected studies. Selection of data for presentation in results was done after all authors agreed to the selection process.

According to well-defined inclusion criteria, data on the prevalence of alexithymia in HD patient samples were analyzed, including their age and duration of dialysis, relationship of alexithymia and other comorbidities, laboratory variables and correlation and the influence of alexithymia mental health factors like depression and quality of life.

Risk of bias on a level of individual study was analyzed with the participation of all authors and after agreement, we excluded negative results of studies based on small samples from further analyses because these studies: had type two statistical errors, did not have an accurate study design according to planned aims, had longitudinal results that did not adequately measure comorbidities as confounding factors or because they which had completed ambitiously performed statistical analyses without an adequate number of participants for that level of evidence.

## 3. Results

**Figure 1:**
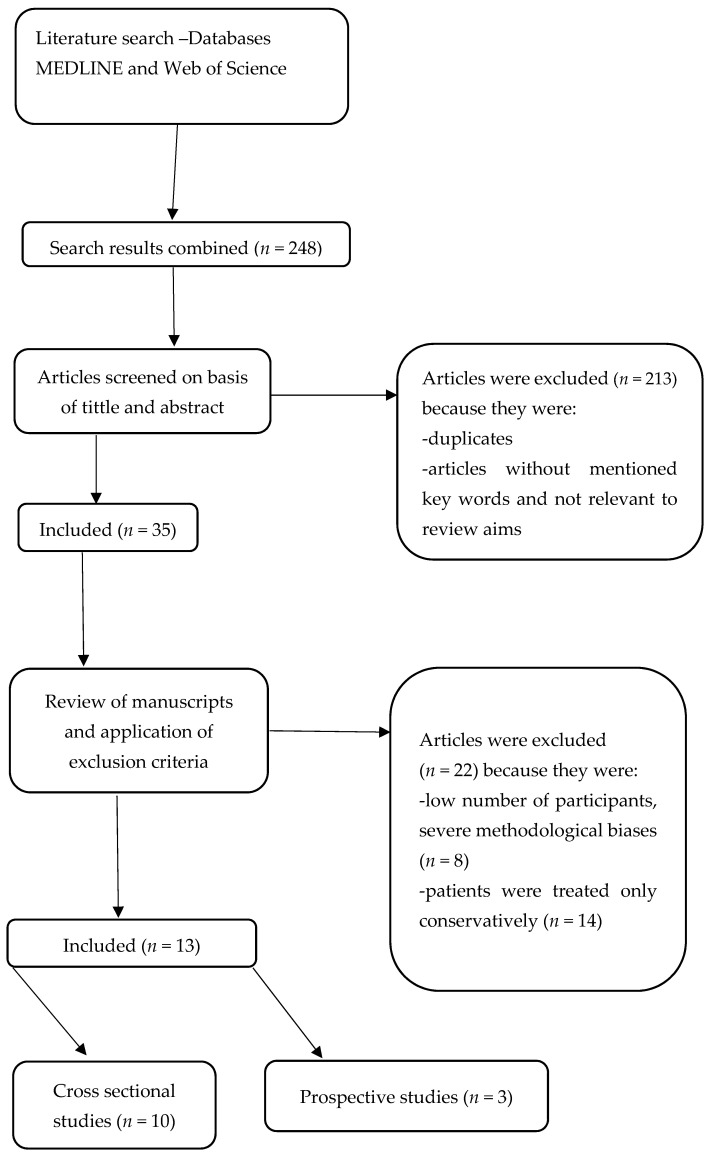
Search and selection protocol of studies.

The results of the preliminary search of the above databases were 248 articles. Articles were excluded based on the above exclusion criteria, as were articles that did not contain the above keywords in their title and abstract and articles that were repeated across different databases. The above process narrowed the selection of literature to 35 articles, of which 13 were included in this literature review. The remaining 22 articles were excluded because the subject of the study was patients who had received transplants or patients undergoing peritoneal dialysis. Of the selected 13 articles, 3 were longitudinal studies and 10 of them were cross-sectional studies (Figure 1).

In the reviewed literature, assessment instruments for personality traits characteristic of alexithymia were self-assessment scales with good internal consistency. However, the downside of all studies is the self-assessment character of the questionnaires, since personality traits characteristic of alexithymia are not easy to identify for an alexithymiac, which likely decreases the actual levels of alexithymia. In studies conducted before and during 1992 and 1993, the “Beth Israel Questionnaire” (BIQ) and “MMPI alexithymia scale” (MMPI) were used, while newer research applied the generally accepted “Toronto Alexithymia Scale 20” (TAS-20) and “Bermond-Vorst Alexithymia Questionnaire” (BVAQ), which include a scale for measuring reduced fantasizing, an alexithymia component that was left out of TAS-20 [17].

Characteristics of individual studies and their limitations in terms of bias at individual study level are presented in a matrix of studies and indicated by date of publication, allowing for better understanding and simpler evaluation of selected studies (Table 1).

The results of the conducted search are factual findings on alexithymia in HD patients, which are grouped into sections on prevalence of alexithymia and its correlation with clinical and psychosocial factors.

### 3.1. Prevalence of Alexithymia in the Population of HD Patients

The prevalence of alexithymia in HD patients ranges from 13.9% to 83% [7,19]. The study conducted by Kojima on a sample of 230 young and otherwise healthy HD respondents for the purpose of observing the impact of basic alexithymia on the five-year mortality rate established that the prevalence of alexithymia was 13.9% [7].

A prospective study conducted by De Santo et al. showed that the prevalence of alexithymia was 83% in the sample of HD respondents with severe secondary hyperparathyroidism [23]. High levels of alexithymia have were established in HD patients with diabetic nephropathy, with a prevalence of about 66.2%, which was higher than in HD patients with nephritis or other causes of end stage renal disease in that study [26]. Results of the study conducted by Pop Jordanova and Polenakovic showed that the levels of secondary alexithymia in HD patients are some of the highest when compared to patients with other somatic disorders; in the above study, the prevalence was 50% in HD patients, which was significantly higher than alexithymia levels measured in the comparable group of patients with carcinoma [21].

Several studies established higher levels of prevalence of alexithymia in HD patients above the age of 60, as well as in HD patients above the age of 65 whose treatment lasted longer in comparison with HD patients of the same age whose hemodialysis treatment had just begun [9,26].

### 3.2. Correlation between Alexithymia, Laboratory Values and Comorbidities in HD Patients

Alexithymia established in the samples of HD patients was correlated with several different laboratory variables and clinical factors.

Tayaz et al. also proved the negative correlation of TAS-20 scores with creatinine levels in HD patients [19]. A negative correlation of alexithymia with dry weight of HD patients, levels of urea after hemodialysis treatment and levels of phosphorus before hemodialysis treatment was found [18].

The “TAS-3” subscale of the “TAS-20” questionnaire, which measures externally oriented thinking has a negative correlation with the values of serum potassium immediately prior to the day of hemodialysis treatment [19]. Also a negative correlation between externally oriented thinking and levels of serum creatinine before hemodialysis treatment as with lower levels of intradialytic weight gains was demonstrated [18,19].

Certain studies proved a higher prevalence of alexithymia in diabetic HD patients compared to other groups of HD patients [26], such as elderly HD patients on hemodialysis for longer periods of time [9,22], and HD patients with uremic pruritus [20], and also proved the correlation between alexithymia and enlargement of the heart expressed as the cardiothoracic ratio [9]. A prospective study of HD patients undergoing a parathyroidectomy described that normalization of hemoglobin levels had a significant impact on the reduction of alexithymia 24 months after the surgery; a decrease in the number of HD patients using high blood pressure medications also had a significant predictive effect [23]. Alexithymia was also identified as a factor that correlates negatively with the physical component of HD patients’ quality of life and the strongest level of proof regarding its effects on somatic health was provided by the fact that alexithymia, controlled for sociodemographic and clinical factors, was identified as the predictor of a 3.62 times higher death rate of HD patients [7]. This argument was made in a longitudinal study with a sample of 230 HD patients in a five-year follow-up period [7].

### 3.3. Alexithymia and HD Patients’ Mental Health

In HD patients with alexithymia, social support has a protective effect on the development of a depressive disorder, noting that at the start of development of a depressive disorder in HD patients, the item “satisfaction with social support” is more relevant, while subsequent worsening of depression symptoms is prevented by the number of individuals providing support to the depressed individual [24]. A mutual causal relationship between alexithymia and depression was also proved in a sample of HD patients, where the decrease of depression was a significant predictor of a decrease in levels of symptoms connected with alexithymia at the end of a two-year observation period of HD patients after parathyreoidectomy [23].

Sinatra et al. established significantly higher levels of depression symptoms and scores of subscales of alexithymia, which led to difficulty describing and identifying feelings that predicted depression positively in patients undergoing hemodialysis treatment for a period shorter than four years [22]. After the above period, HD patients accept limitations imposed by hemodialysis treatment, and they are considered to be eligible for a kidney transplant. However, traits connected with alexithymia may decrease the motivation of HD patients to receive a kidney transplant, and they may decrease the patients’ reasons to live, which is especially evident in the group of elderly HD patients [27].

High levels of alexithymia are also correlated with a higher prevalence of bulimia in the sample of HD patients, especially if nephropathy is of a diabetic etiology [26].

The fact that HD patients with alexithymia show lower levels of conflict and extroversion in comparison with HD patients without alexithymia and members of their own family, although there are numerous objective reasons for emergence of conflicts, is likewise interesting [28].

## 4. Discussion

The prevalence of alexithymia in HD patients, which ranges from 13% to 83%, is significantly higher in comparison with alexithymia levels in the general population, where the prevalence of alexithymia ranges from 4% to 13% [29]. The only study that showed the prevalence of alexithymia (13%) in HD patients as similar to its prevalence in the general population was the study conducted by Kojima et al., most likely because the sample included mainly younger HD patients with a lower number of comorbidities. They were chosen based on their physical ability to arrive independently to the institution where the research was carried out [7]. Differences in the high prevalence of alexithymia levels in HD patients result from clinical differences in the samples of participating respondents, such as diabetes, severe secondary hyperparathyroidism and similar conditions.

Advanced age is a factor that is related to higher levels of alexithymia in the general population as well, and a potential reason for that are functional changes to the brain’s frontal lobe. Persons over the age of 65 with no comorbidities or signs of dementia show significantly higher levels of alexithymia in comparison with younger persons, and the greatest contribution to high levels of alexithymia is given by reduced cognitive function of the right frontal lobe, visual memory and nonverbal intelligence [30]. Due to frequent hypotensive episodes caused by an improperly set ultrafiltration rate, long-term hemodialysis treatment could cause damage to the above mentioned sections of the right frontal lobe, thus causing an increase in alexithymia [2]. Since HD patients of advanced age who have been undergoing dialysis treatment for a while have significantly higher levels of alexithymia when compared to patients of the same age who are only starting their hemodialysis treatment, it can be concluded that other factors, in addition to age, likewise cause alexithymia levels to increase [9]. The duration of hemodialysis treatment in the observed group could be one such factor, though the conclusion would be more appropriate if the studies were longitudinal [9].

A low socioeconomic status of HD patients may be one of the causes of extremely high levels of alexithymia, since working-age HD patients regularly have reduced physical and mental capacity, while the time-consuming nature of treatment imposes additional restrictions on finding adequate employment [31]. Persons with lower socioeconomic status are permanently focused on looking for employment and material assets, which orients their thoughts toward external factors rather than intrapsychic processes and emotions, thus contributing to the development of alexithymia [32].

Alexithymia is a predisposing factor that leads to the development of mental and somatic disorders; the significant role that this construct plays in functional gastrointestinal disorders, but also eating disorders such as bulimia and anorexia has been confirmed [33,34]. However, in HD patients, there is a different side to the mutual correlation between alexithymia and somatic health, since the treatment process likewise leads to an increase in the inability to distinguish between somatic sensations and emotions; the severity of comorbidities additionally contributes to such an increase [23]. Since HD patients are exposed to the dietary restrictions in terms of specific foods and amount of fluid intake, and to constant physical and mental stress and disturbing scenes in hemodialysis units, it is possible that alexithymia levels increase as a result of using denial as a defense mechanism, which contributes to the preservation of mental health in the short term [3,35]. Denial of physical and mental stress arising from hemodialysis treatment may lead to denial of dietary and treatment recommendations, which is a direct cause of inadequate fluid intake in HD patients [11]. There is also a telling difference between alexithymia factors in regard to seeking medical assistance; the factor “difficulty identifying feelings” is connected to more frequent requests for medical assistance, while “externally oriented thinking” is connected to approaching preventive medicine programs [36]. The above may explain the correlation between the latter subscale and low levels of serum potassium, which is completely unexpected considering the low diet control of alexithymiacs.

We have already mentioned that an increase in levels of alexithymia in HD patients occurs through a synergic connectedness of physical and mental limitations and various comorbidities arising from chronic kidney failure and its cause. Hemodialysis treatment may be seen as a repetition of physical and mental trauma due to frequent complications, muscle cramps, hypotension and discomfort [31,32]. Long-lasting stress caused by traumas that physically and mentally endanger the individual, such as rape or severe burns, leads to the development of alexithymia in the general population, which continues to be present even after physical damage has healed [32]. Restrictions, endangerment and stress that arise from the need to treat diabetes or severe hyperparathyroidism further exacerbate the levels of alexithymia in HD patients and likewise represent repeated traumas [37]. Patients with diabetes must have a tailored diet plan and regularly control blood glucose levels; they show extremely high levels of alexithymia even before commencing hemodialysis treatment [26]. Hemodialysis treatment in this group of patients leads to an even greater increase in alexithymia, which once again confirms the fact that alexithymia is not only the cause of somatic disorders, but also a consequence thereof. Alexithymia and somatic disorders in HD patients close the positive feedback loop, where difficulties in differentiating between somatic problems and emotions lead to an increase in intensity of physical problems, which are thus amplified with time. HD patients become incapable of recognizing negative affect—they attribute it to somatic problems, which arises from the fact that when alexithymiacs experience negative mood states, there is increased self-assessment of bodily sensations [3]. Unrecognized negative affect likely arises from conflicts on a mental level, which are caused by failure to accept limitations imposed by hemodialysis treatment; for that reason, HD patients convert everyday frustrations into severe uremic pruritus [20,38].

The inability of HD patients to accept and identify negative affect leads to the development of a depressive disorder, which again has a negative impact on the quality of life of HD patients and the emergence of comorbidities [23]. Although there were suspicions in that regard, it has been proven that alexithymia is a construct separate from depression. Using a large sample of HD respondents, Oogai et al. conducted a study in which HD patients filled out a questionnaire for measuring depression and alexithymia, in which the items were grouped into two separate constructs through factor analysis, with no overlap between the two [25].

Unlike newer studies, in which alexithymia was measured using the TAS-20 questionnaire and where there was a clear positive correlation between alexithymia and depression, results of older studies (MMPI, BIQ) refer to a negative correlation between these two separate nosological terms [9]. However, due to psychometric deficiencies of the MMPI questionnaire, precedence should be given to the findings that include a positive correlation between alexithymia and depression [7,32]. The higher the levels of alexithymia in HD patients, the greater the risk of developing a depressive disorder [24]. A mitigating circumstance for HD patients is the fact that, unlike in the general population, support that HD patients receive from medical staff and other HD patients decreases depression symptoms [39].

Kojima et al. established that, independent of depression levels, alexithymia is the predictor of a 3.62 times higher death rate in HD patients [7]. The above is likely realized through the tendency of alexithymiacs for consuming unhealthy food, for alcoholism, smoking and a sedentary lifestyle, all of which are factors that increase the risk of cardiovascular diseases [40,41]. Alexithymia in clinical research is correlated to a significantly higher incidence of arteriosclerosis of the coronary blood vessels, and clinical research has shown that levels of alexithymia are significantly higher in patients with 75% coronary lumen stenosis in comparison with patients with any coronary blood vessel stenosis lower than 25% [42]. The fact that cardiovascular diseases are the most common cause of death in HD patients supports the above [12].

Given the findings on the prevalence of alexithymia in the HD patient population, the question arises whether it is possible to influence the prevention and treatment of alexithymia in HD patients. To that end, we propose a number of methods. One of them would be more adequate treatment of all other somatic diseases that HD patients suffer from, with a focus on better diabetes control, better secondary hyperparathyroidism treatment and treatment of mental disorders such as depression. Given that the aforementioned diseases are associated with higher levels of alexithymia and that their better management would likely result in lower levels of alexithymia, the vicious circle in which severe somatic and mental states of HD patients lead to decreased cognitive emotion assessment would be broken. The results in the available literature demonstrate that treating depression and eating disorders such as bulimia and anorexia result in decreased alexithymia levels, even when psychotherapeutic methods are not specifically aimed at alexithymia [43]. The only study in the reviewed literature dealing with direct psychotherapeutic treatment of alexithymia demonstrated a remarkable effect of such treatment on the reduction of alexithymia in a sample of 20 patients with coronary heart disease, which was associated with fewer reports of cardiovascular symptoms, hospitalizations and deaths over a two-year follow-up period [44]. We believe that similar group psychotherapy, in which HD patients are encouraged to practice identifying their own feelings and the feelings of others in the group, describing internal emotional states and using their imagination, could result in a similar decrease in alexithymia levels.

The results of this literature review should be taken with a grain of salt, because the studies included in the review have certain flaws. A number of older studies included in the review had a small number of respondents, which is likely due to the fact that hemodialysis treatment was limited by age group at the time, resulting in a generally low number of HD patients. Still, in the absence of findings about alexithymia, we included the above studies in our review because there were significant differences in the observed variables, even for such a small sample. Taking the type 2 error rules into account, it is likely that such differences would exist even if the number of respondents was larger. Furthermore, older studies measured alexithymia using the MMPI scale, which has poor psychometric properties; such articles thus considered the results obtained using other questionnaires as well. Thirdly, all the results were obtained using self-assessment scales, which is questionable, because in the questionnaires, the respondents are supposed to report on their emotions and bodily sensations despite the fact that alexithymia is, in fact, a concept that refers to a lack of insight into one’s emotions and bodily sensations.

## 5. Conclusions

Alexithymia is a factor that negatively affects mental health and quality of life of HD patients [45]. Although it has a significant impact on the development and worsening of depressive symptoms, alexithymia is a distinct nosological factor completely different from depression in that it represents the inability to distinguish one’s feelings from bodily sensations and inability to describe one’s feelings to others. Alexithymia is more prominent in HD patients with severe comorbidities; it indirectly indicates a more severe somatic condition and may lead to poor communication due to difficulties in identifying one’s bodily needs. HD patients with alexithymia may frequently fatigue medical staff who are unable to fully assess the validity of such patients’ complaints and requests, which they are required to do in the long run. The “externally oriented thinking” subscale of alexithymia might be partially useful, seeing as patients with high scores on said subscale have significantly lower levels of serum potassium prior to the next hemodialysis treatment [18].

Given the small body and low strength of evidence from previous research on alexithymia in HD patients, future studies should examine the association and causality between alexithymia and laboratory electrolyte values (potassium, phosphorus), hypervolemia and systemic inflammation, other somatic comorbidities as well as mental health factors, to determine the mechanisms through which alexithymia has such a strong impact on HD patient mortality [7]. Future studies should also be based on a more objective, evaluative measurement of alexithymia, with an objective evaluation by a clinical psychologist or psychiatrist.

## Figures and Tables

**Table 1:** Matrix of relevant literature. Hemodialysis (HD), Cross-sectional design (CSD), Prospective multicentric design (PMD), Prospective design (PD); Center for Epidemiological Study Depression Scale (CES-D).

Author (Year)	Aims of the Study	Study Design/Methods	Number of Patients (HD)	Strenghts	Limits
Akyüz, O. et al. (2021); [18]	Investigation of the relationship between biochemical variables, alexithymia and stress levels.	CSD; TAS 20	51	The sample of patients correctly represents population of HD patients in relation to age and comorbidities	Single center study with a small patient sample; there was retrospective insight in biochemical values
Tayaz E. (2019); [19]	Association of alexithymia with laboratory values in HD patients.	CSD; TAS 20	72	Single center study	CSD, small number of participants, single measurement of laboratory values.
Heisig M. et al. (2016); [20]	Comparison of levels of alexithymia in HD patients with and without uraemic pruritus.	CSD; BVAQ	42 of 90 patients had pruritus	Multicentric study	CSD
Pop Jordanova et al. (2014); [21]	Evaluating the effects of age, duration of dialysis and gender on levels of alexithymia in HD patients.	CSD; TAS 20	230 HD patients	Multicentric study, large sample of patients	
Sinatra M. et al. (2011); [22]	Comparison of depression, alexithymia, social support and quality of life between HD patients dialyzed for less than 4 years and those dialyzed for more than 4 years.	CSD; TAS 20	103 HD patients, 101 patient in control group		CSD, self-reported questionnaires and relatively old participants
De Santo et al. (2010); [23]	Comparison of alexithymia levels in HD patients requiring parathyroidectomy (PTX) and those not requiring PTX at baseline and 24 months following PTX.	PD; TAS 20	40 HD patients needing PTX, 80 HD patients not needing PTX	Prospective design of a study enables establishment of causality	
Kojima M. et al. (2010); [7]	Analyzing the effect of alexithymia, depression and laboratory values on five-year mortality among HD patients.	PMD; TAS 20	230 HD patients	Prospective study; Comorbidities and laboratory values at the baseline and excludes confounders	Self-reported scales; small sample size for Cox regression analysis
Kojima M. et al. (2007); [24]	Assesing the effect of baseline alexithymia, depression and percieved social support on deterioration of depression after a 6-months follow up period in HD patients.	PMD; TAS 20	229 HD patients	PMD	Self-reported questionnaires, depression is defined through result of BDI-II questionnaire, sample is grouped in small area of city
Oogai Y. et al. (2003); [25]	Examination of overlapping of alexithymia and depression constructs in HD patients.	CSD; TAS 20, CES-D	507 HD patients	Multicentric study, includes a large number of participants	
Fukunishi I. (1997); [26]	Comparison of prevalence of alexithymia in HD patients in terms of etiology of ESRD and presence of bulimia nervosa.	CSD; TAS 20	312 HD patients (130 in diabetic group), 16 patients had bulimia nervosa	High number of patients in each group enables good statistical strength	Single center study
Fukunishi I. (1993); [27]	Determining the effect of alexithymia on satisfaction with HD, sense of purpose in life and desire for kidney transplantation.	CSD; BIQ	191 HD patient younger of 59 years and 84 patients who are 60 years old or older	Large number of patients	Single center study and cross-sectional design
Fukunishi I. et al. (1992); [3]	Comparing alexithymic HD patients and their family members to those without alexithymia on family environment subscales.	CSD; BIQ, TAS 20	35 HD patients and 35 of their family members		A single center study with a small number of participants and cross-sectional design does not allow establishment of causality
Fukunishi I. (1989); [9]	Comparing the levels of alexithymia in patients over 65 on long term HD (S1 group) and HD patients 65 who just started hemodialysis (S2 group)	CSD; BIQ	50 HD patients in S1 group and S2 group, control groups (C1-Hd patients younger than 65 years, C2 groups) with 50 patients each	There is no causality, because of a small number of patients in S1 group stratified by comorbidities, leading to a high possibility of a type 2 statistical error	

## Data Availability

The data presented in this study are openly available in repositories at [doi]; links are given near each reference. Data are available in a publicly accessible repository that does not issue DOIs. Publicly available datasets were analyzed in this study. This data can be found at the link given near each specific reference.
